# Drought effects on tree growth, water use efficiency, vulnerability and canopy health of *Quercus variabilis-Robinia pseudoacacia* mixed plantation

**DOI:** 10.3389/fpls.2022.1018405

**Published:** 2022-10-12

**Authors:** HanSen Jia, ChongFan Guan, JinSong Zhang, ChunXia He, ChangJun Yin, Ping Meng

**Affiliations:** ^1^ Key Laboratory of Tree Breeding and Cultivation, National Forestry and Grassland Administration, Research Institute of Forestry, Chinese Academy of Forestry, Beijing, China; ^2^ Collaborative Innovation Center of Sustainable Forestry in Southern China, Nanjing Forest University, Nanjing, China; ^3^ Henan Xiaolangdi Earth Critical Zone National Research Station on the Middle Yellow River, Jiyuan, China

**Keywords:** drought stress, radial growth, resistance, stable isotopes, canopy die-back

## Abstract

Drought-induced forest canopy die-back and tree mortality have been commonly recorded in the lithoid mountainous regions of northern China. However, the capacity of trees to regulate their carbon and water balance in response to drought remains inadequately understood. We measured tree growth, intrinsic water use efficiency (iWUE), vulnerability, and canopy health during drought events using dendrochronology, C isotope measurements, and a tree canopy health survey in a mixed plantation of *Quercus variabilis* and *Robinia pseudoacacia*. Resistance (Rt), recovery (Rc), resilience (Rs), and increased amplitude in iWUE compared to the indices 3 years before drought (iWUEr) were calculated for each species across the dominant tree (D), co-dominant tree (CD), and suppressed tree (S). Our results revealed that D and CD showed lower Rt, higher Rc, and higher iWUEr than S. After exposure to multiple sequential drought events, *Q. variabilis* showed an increasing trend in Rt, and *R. pseudoacacia* showed a decreasing trend in Rc. *R. pseudoacacia* exhibited a more conservative strategy towards drought, resulting in a negative S_Rt-iWUEr_ (slope of the linear model fitted to capture the trend between Rt and iWUEr) during drought events than *Q. variabilis*. For individual trees, lower Rc or positive S_Rt-iWUEr_ *Q. variabilis* and negative S_Rt-iWUEr_ *R. pseudoacacia* were more susceptible to canopy die-back. In conclusion, our study offers a new perspective for improved management practices in the design of silvicultural actions for forestry plantations in lithoid mountainous areas with increasing drought risk.

## Introduction

Since the 1970s, China has implemented an ecosystem restoration program to repair vulnerable ecosystems and restore trees in previously degraded ecosystems (Cao et al., 2011). However, weak growth rates and tree mortality have arisen in large-scale plantations, even though many tree species have been chosen for drought tolerance (Wu et al., 2004; Sun et al., 2018b; Zhao et al., 2018). For these plantations, despite major progress in understanding how water deficits affect functioning (Wang et al., 2017; Sun et al., 2018a), the relationship between functional indices and species survival under extreme drought remains poorly understood and limits our ability to adequately predict tree mortality risk accompanied by continuing warming and aridification in the future.

Recent syntheses of drought physiology have highlighted the need to consider the capacity of trees to regulate their carbon and water balances under drought conditions (Mitchell et al., 2013; Gentilesca et al., 2021). These frameworks suggest that carbon starvation (Sevanto et al., 2014) or hydraulic (Grote et al., 2016) failure may be responsible for the drought-induced mortality in trees. Also, it has been observed that drought stress changes with selection of tree species or tree species mixtures (Pretzsch et al., 2013; Merlin et al., 2015). Different species growing in the same soil water status may perform differently in a mixed forest, and drought will likely disturb the current equilibrium between the co-existing species (Fekedulegn et al., 2003; Tognetti et al., 2007; Jucker et al., 2014). For example, in mixed plantations on the Loess Plateau, top shoot die-back and mortality are frequent in *Populus hopeinsis* and *Robinia pseudoacacia*. However, other species such as *Ulmus pumila* rarely have similar difficulties (Zhang and Shang, 2002). The reasons for these species-specific differences in response to drought are still not thoroughly understood. In addition to these drivers, the presence of various individual tree social statuses within a stand adds another layer of complexity to the tree’s response to drought (Merlin et al., 2015). This social position suggests a disparity in the accessibility of resources such as water, nutrients, and light, which results in differences in the reactions to severe droughts (Trouvé et al., 2017). Consequently, some studies have comprehensively discussed the effect of water stress on the growth of deciduous trees of various social statuses. However, these findings are inconsistent (Liu and Muller, 1993; He et al., 2005). Therefore, it is critical to incorporate both species and social status into the stand as explanatory factors when evaluating the carbon and water balance responses of mixed plantations to drought.

In particular, tree growth resilience indices and intrinsic water-use efficiency (iWUE) are important indicators for the response analyses of carbon and water balance to drought (Forner et al., 2018; Sun et al., 2018a; Nolan et al., 2021; Zhang et al., 2022). Forest stability reactions during drought events are typically categorized as having either immediate or delayed effects, representing their ability to maintain functions during and after drought events (Trouvé et al., 2017; Zas et al., 2020). iWUE is defined as the net photosynthetic rate to stomatal conductance, which evaluates the water loss through the stomata. The trade-off between carbon capture and water loss is not a serious conflict in favorable climate conditions, resulting in a slight increase in tree growth when iWUE increases (Guerrieri et al., 2019; Lu et al., 2019). Under extreme drought events, the increase in iWUE is mainly induced by closing stomata to save water and maintain their leaf water potential within a safe range at the expense of reduced carbon uptake (Martin-Benito et al., 2017). In this case, the growth resistance of the trees will decrease or remain constant, which has been reported in the recent literature on dead trees under controlled environmental conditions (Sun et al., 2018b; Zadworny et al., 2019). Previous research has provided insights into tree growth and iWUE responses to drought in North China, although the results are inconsistent (Wu et al., 2015; Sun et al., 2018b; Song et al., 2019). However, few empirical studies have evaluated the effectiveness of these indicators for predicting the risk of drought-induced mortality.

In this study, we used dendrochronology and carbon isotopes to understand the growth resilience indices and iWUE for *Q. variabilis* and *R. pseudoacacia* across three crown classes. We related growth resilience indices, iWUE, with the current canopy health score, a proxy for vulnerability to canopy die-back, to assess the relationships between these indicators and drought-induced mortality. The following two questions were the focus of our investigation: (1) How did growth resilience indices and iWUE vary between species, crown classes, and drought events? (2) Which post-drought resilience and iWUE indices influence current canopy die-back?

## Materials and methods

### Study area

**Figure 1:**
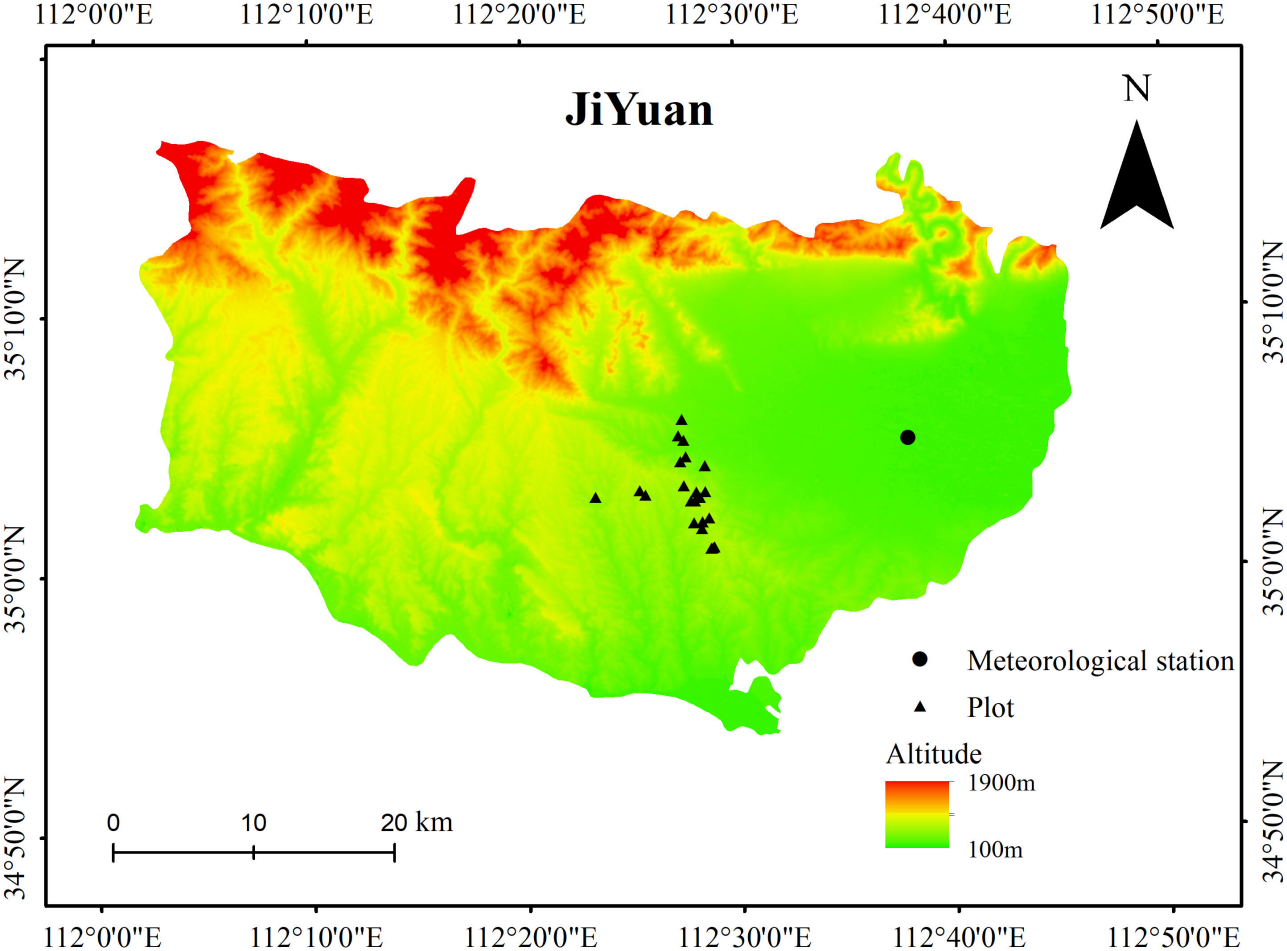
The location of the plots and the nearby meteorological stations.

**Figure 2:**
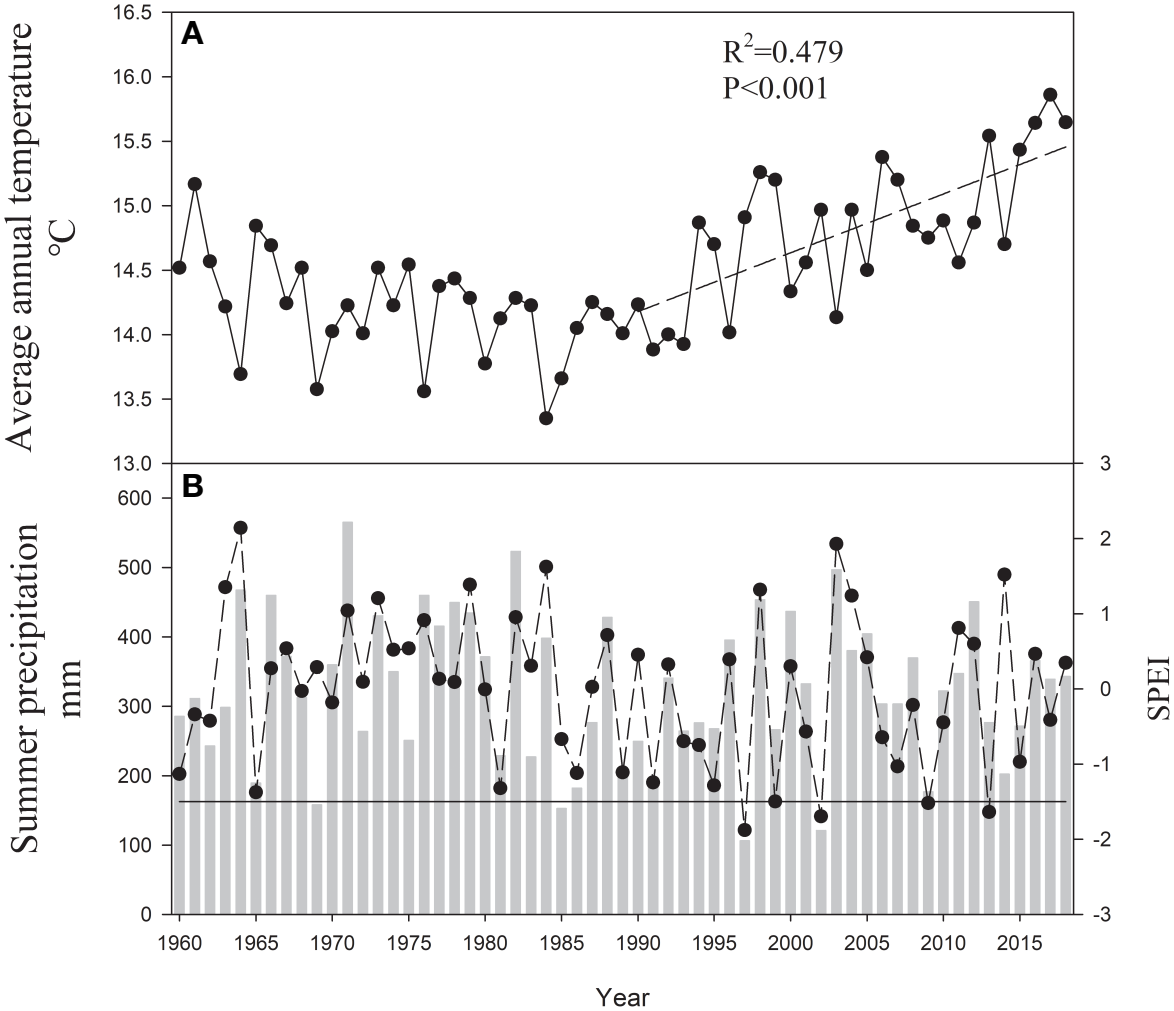
**(A)** Average annual temperature (T), the dotted line indicates the trends considering T for the 1990–2018 period; **(B)** summer (June to August) precipitation (P) and SPEI calculated from October of the previous year to September of the current year (SPEI12sep) – dotted line for the 1960–2018 period. The dark line indicates the SPEI threshold of -1.5 (indicative of severe drought).

This study was conducted in a young mixed forest at a height of approximately 410 m above sea level in the lithoid hilly region of North China (35°02’ N, 112°28’ E) (**Figure 1**). Warm-temperate continental monsoon weather prevailed at the site over the course of 50 years, with a mean annual temperature of 14.5°C and mean annual precipitation of 609.3 mm. In the study region, there has been a significant increase in the annual mean temperature over the last 30 years (**Figure 2A**). The geological substrate is composed of limestone. The soil at the site was 40–50 cm deep and classified as brown loam. The sites were covered by 46-year-old mixed deciduous *Q. variabilis* and *R. pseudoacacia* trees.

### Climatic data

Monthly mean temperature and total precipitation data were obtained from the Jiyuan County Meteorological Station (35°5’ N, 112°38’ E, 10 km from the site; **Figure 1**). We obtained the standardized precipitation evapotranspiration index (SPEI) calculated from October of the previous year to September of the current year (SPEI12sep) to evaluate drought severity with the SPEI program (Vicente-Serrano et al., 2010). This methodological approach avoids climatic parameters in the months following stem growth (Conte et al., 2018). Positive SPEI values imply wet conditions, whereas negative values indicate dry conditions. We selected three drought years (years with SPEI12sep< −1.5: 2002, 2009, and 2013) for the period 1960–2018 (**Figure 2B**).

### Field sampling and dendrochronological methods

A forest survey was conducted in August 2019 by establishing 23 inventory plots (30 × 30 m). Geographic coordinates and characteristics of these plots are provided in **Supplementary Table S1**. In each plot, trees with a diameter at breast height (DBH) ≥ 4.0 cm were permanently marked, measured (DBH, height X–Y coordinates), and recorded (species and crown class). The crown classes were defined as dominant (D), co-dominant (CD), and suppressed (S). In the present study, D trees had crowns extending above the main canopy, CD trees had crowns making up the main canopy, and S trees had crowns extending into the lower portion of the main canopy (Seidel et al., 2011).

Fifty to sixty trees were selected from each plot. In total, 1265 trees were sampled (799 *Q. variabilis*; 466 *R. pseudoacacia*). Two complete cores (90° from one another) were collected per sample tree perpendicular to a height of 1.3 m with standard 5.15-mm increment borers (Häglof, Långsele, Sweden). The cores were air-dried and sanded with papers of progressively finer grains until tree rings were clearly visible. Tree rings were dated under a stereomicroscope (Leica, Germany), and tree-ring width was measured using a LINTAB measuring device (Frank Rinn, Heidelberg, Germany, resolution: 0.01 mm). Cross-dating was further validated using COFECHA v11 (Holmes, 1983).

Several statistics commonly used in dendrochronology were calculated to assess the quality of the tree-ring width series using the dplR package (Bunn, 2008): first-order autocorrelation (AC) of raw width data (Fritts, 1976), mean sensitivity (MS) of indexed growth values, mean correlation (Rbt) between trees (Briffa, 1990), and expressed population signal (EPS) (Wigley et al., 1984), which measures the statistical quality of the mean site chronology as compared with a perfect, infinitely replicated chronology (**Table 1**).

**Table 1:** Chronology statistics in dominant trees (D), co-dominant trees (CD) and suppressed trees (S) for sampled Q. variabilis and R. pseudoacacia.

Species	Crown classes	Period	No. of trees/cores	MS	Rbt	AC	EPS
*Q. variabilis*	D	1972-2018	232/462	0.332	0.352	0.412	0.983
	CD	1972-2018	302/604	0.278	0.332	0.337	0.982
	S	1979-2018	265/530	0.251	0.272	0.321	0.912
*R. pseudoacacia*	D	1972-2018	133/266	0.274	0.343	0.493	0.986
	CD	1974-2018	184/368	0.265	0.277	0.389	0.978
	S	1982-2018	149/298	0.262	0.231	0.289	0.934

Rbt, mean between-trees correlation; MS, mean sensitivity; AC, first-order autocorrelation; EPS, expressed population signal.

The past diameters were extrapolated using the tree-ring widths of the cores. A proportional approach was used to reduce the impact of eccentricity on the growth increment (Bakker, 2005). The tendency caused by the geometrical limitation of adding a volume of wood to a stem with increasing radius was corrected by translating tree-ring widths into basal area increments (BAIs) as follows:


(1) $$\text{BAI}\;=\;\pi (R^{2}n_{-}R^{2}n_{-}1)$$

where R is the tree radius and n is the year of tree-ring formation.

### Growth resilience indices

To evaluate growth resistance and resilience to each drought event, we calculated three indices for each species and crown class using BAI data: resistance (R_t_), recovery (R_c_), and resilience (R_s_) (Lloret et al., 2011).


(2) $$\text{Resistance}{\;}_{\text{Rt}}={\mathrm{BA}}_{\text{ID}}/\text{BA}{\text{I}}_{\text{pre}}$$

(3) $$\text{Recovery}{\;}_{\text{Rc}}=\text{BA}{\text{I}}_{\text{post}}/{\mathrm{BA}}_{\text{ID}}$$

(4) $$\text{Resilience}{\;}_{\text{Rs}}=\text{BA}{\text{I}}_{\text{post}}/\text{BA}{\text{I}}_{\text{pre}}$$

where BAI_D_ is the mean BAI during a drought event and BAI_pre_ and BAI_post_ are the mean BAIs in the 3 years before or after the drought event.

### Water use efficiency analysis

After cross-dating, several similar-age tree cores of each crown class were manually separated into slivers using a dissecting scope and razor blade. A total of 135 trees were selected (68 *Q. variabilis* and 67 *R. pseudoacacia*). To estimate the effects of the iWUE response to drought, we analyzed iWUE in drought events (iWUE_D_) and the average iWUE in the 3 years before the drought event (iWUE_B_). The wood of each drought event and 3 years before each drought event was homogenized into a sample. All samples were ground into powder using a ball mill instrument. Whole wood samples were subjected to isotopic analysis as recent research has demonstrated that whole wood can be used instead of a-cellulose in short-term eco-physiological and dendrochronological studies to measure stable C isotopes (Au and Tardif, 2009). Approximately 3–4 mg of each sample (dried for 24 h in an oven at 60°C) in a tin cup was completely burnt to gas using a TOC Element Analyzer (Elementar Analysensysteme GmbH. Hanau, Germany). It was then injected into a carbon dioxide isotope analyzer (CCIA-38-EP, Los Gatos Research, USA) to determine *δ* ^13^C. Three subsamples were randomly selected from each mixed sample, each of which was taken twice. The bi-weight method was then used to average the six values from each mixed sample into a single value. The *δ* ^13^C values were expressed using the Vienna Pee Dee Belemnite scale.

iWUE (μmol mol^−1^) was calculated according to standard methodologies and formulae was calculated using (Farquhar and Richards, 1984):


(5) $$\text{iWUE}=\text{A}/{\text{gs}}_{=}{\text{Ca}}_{\times }[(1{-}_{\text{Ci}}/{\text{C}}_{a})\;1.6]$$

where A is the rate of CO_2_ assimilation by the leaves, g_s_ is the rate of leaf stomatal conductance, Ci is the leaf intercellular CO_2_ concentration and C_a_ is. atmospheric CO_2_ concentration (Ca). To determine Ci/Ca, we used the equation 6 (Farquhar et al., 1982):


(6) $${\text{Ci}}_{=}\text{ A}/{\text{gs}}_{=}{\;}_{\text{Ca}}\times \left[\left(\delta^{13}{\text{C}}_{\text{tree}}-\delta^{13}{\text{C}}_{\text{atm}}+1)/(\text{b}-\text{a}\right)\right]$$

where *δ* ^13^C_tree_ represents *δ* ^13^ measured from tree rings; *δ* ^13^C_atm_ is the value for the atmospheric value obtained from the Earth System Research Laboratory of the U.S. National Oceanic and Atmospheric Administration. a is the discrimination due to diffusion of ^13^CO_2_ through stomata (a = 4.4‰) and b is fractionation discrimination by Rubisco against ^13^CO_2_ (b = 27‰).

To evaluate the degree of change in iWUE during drought events, we calculated the ratio of iWUE_D_ and iWUE_B_ (iWUEr).

### Canopy health

For each sample tree, health condition was evaluated by the canopy health score described previously (Stone et al., 2008; Nolan et al., 2021) based on visual estimates in August 2019, which summed five crown attribute scores (scale of 1 to 5): crown size, crown density, dead branches, crown epicormic growth, and leaf discoloration. Additional details are presented in **Supplementary Table S2**. Scores ranged from 0 for trees with no leaves left to 25 for healthy trees.

### Data analysis

The differences in Rt, Rc, Rs, iWUE, and canopy health score among crown classes of *Q. variabilis* and *R. pseudoacacia* were analyzed using the least significant difference (LSD) test. Prior to statistical analysis, we checked all indices for normality and homoscedasticity, and statistical significance was set at *p*< 0.05.

To test the relationship between radial growth and iWUE during the three drought events, Rt was linearly regressed against iWUEr for each crown class. In addition, a linear model was fitted to capture the trend between Rt and iWUEr for each sample tree, with the slope of this model (S_Rt-iWUEr_) indicating the magnitude of the growth reduction with an increase in iWUE during the three drought events. We evaluated the tree canopy health score as the response variable in linear regressions against the growth resilience indices, iWUEr and S_Rt-iWUEr._

R 4.1.3 was used to conduct all statistical analyses (Team R C, 2013).

## Results

The EPS values were all greater than 0.85 in every instance to suggesting the qualities of chronologies were sufficient good (**Table 1**). The Rbt, MS, and EPS values were higher for D and CD than for S.

### Radial growth analysis

**Figure 3:**
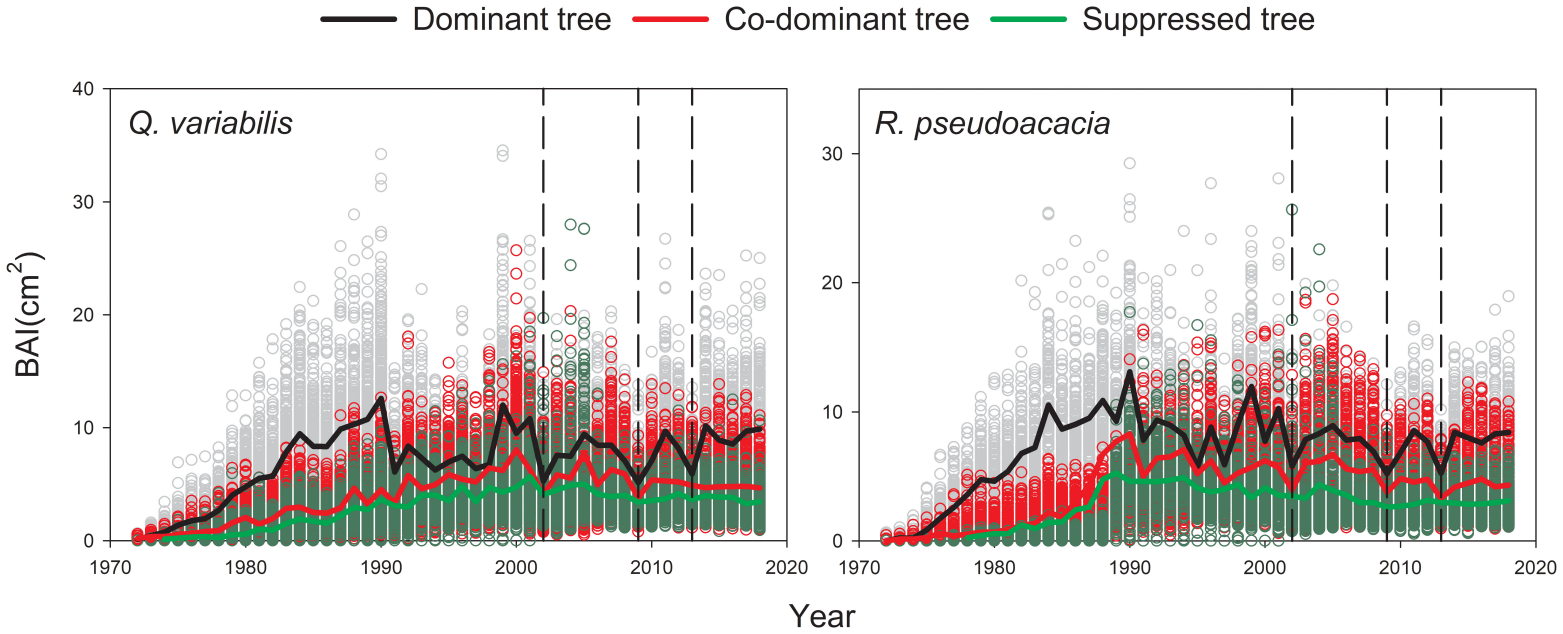
Mean curves of basal area increment (BAI) chronologies of *Q. variabilis* and *R. pseudoacacia* for three canopy classes. The black dotted line corresponds to 2002, 2009, and 2013 drought.

*Q. variabilis* and *R. pseudoacacia* for the three canopy classes had a similar pattern in their BAIs: a steady increase during the first 20 years followed by a substantial and significant decrease during the three drought events (**Figure 3**). A significant negative trend of BAI was observed in suppressed *R. pseudoacacia* (−1.5% year^-1^, *p<*0.01) after the first 20 years, with the trends also being slightly negative but not significant for other types of trees. *Q. variabilis* and *R. pseudoacacia* had lower average BAI values in S than in D and CD.

### Resistance, recovery and resilience to drought

**Figure 4:**
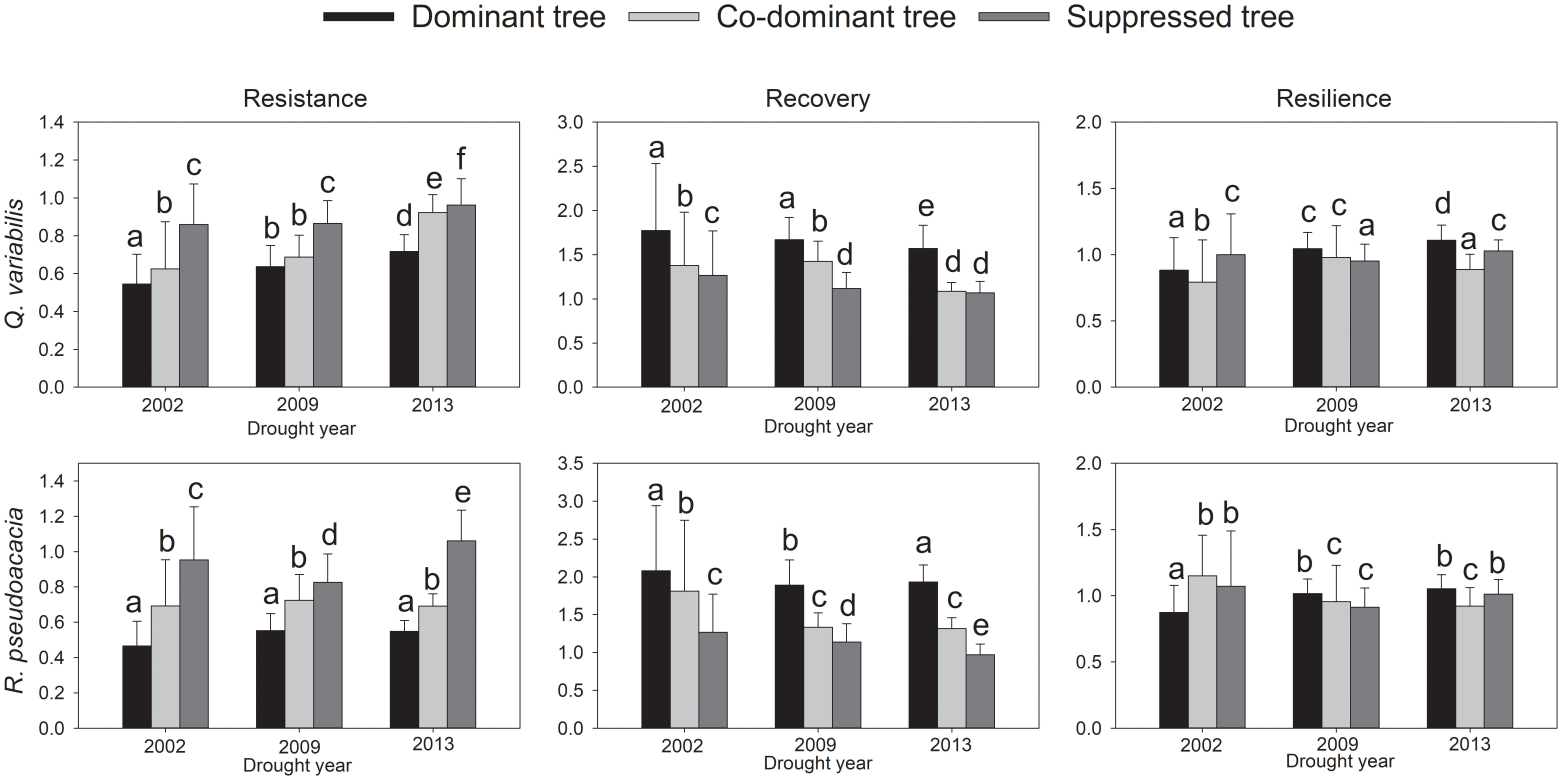
Mean resistance (Rt), recovery (Rc), resilience (Rs) calculated for *Q. variabilis* and *R. pseudoacacia* of three canopy classes during drought events. Different letters indicate significant difference (p< 0.05) in drought responses between three canopy classes.

For *Q. variabilis* and *R. pseudoacacia*, in terms of drought Rt and Rc, significant differences in responses were observed for each drought event between the three canopy classes. The order of the Rt values was S > CD > D, and the order of the Rc values was D > CD > S (**Figure 4**). With the increase in drought events experienced by trees, the Rt of *Q. variabilis* in each canopy class showed an increasing trend, and the Rc of *R. pseudoacacia* in each canopy class showed a decreasing trend. During the 2002 drought event, D was less resilient than S. On the other hand, S was less resilient than D during the 2009 and 2013 drought events.

### iWUE responses to drought

The iWUE of both species significantly decreased in response to drought in relation to the iWUE of the three years preceding drought events (**Table 2**). For the average iWUEr of the three drought events between the three canopy classes, the order of *Q. variabilis* was D (1.21) > CD (1.15) > S (1.11) and that of *R. pseudoacacia* was CD (1.22) > D (1.15) > S (1.10).

**Table 2:** Mean values of iWUE for each species, canopy class.

Period	iWUE (umol·mol^-1^)					
	*Q. variabilis*			*R. pseudoacacia*		
	D	CD	S	D	CD	S
1999-2001	104.58a	103.33a	91.35a	106.64a	97.59a	96.09a
2002	131.93b	123.57b	106.24b	133.54b	119.19b	111.60b
2006-2008	110.02a	118.90b	107.89b	124.11c	115.52b	113.42b
2009	139.66b	136.94c	117.66c	136.34b	140.39c	123.50c
2010-2012	123.38c	125.26b	112.69c	129.35c	121.44b	118.44c
2013	137.56b	137.43c	120.82d	140.50d	145.27d	126.89d

For the same species, different lower case letters indicate significant difference (p< 0.05) of same period between three canopy classes.

### Relationships between Rt and iWUEr

**Figure 5:**
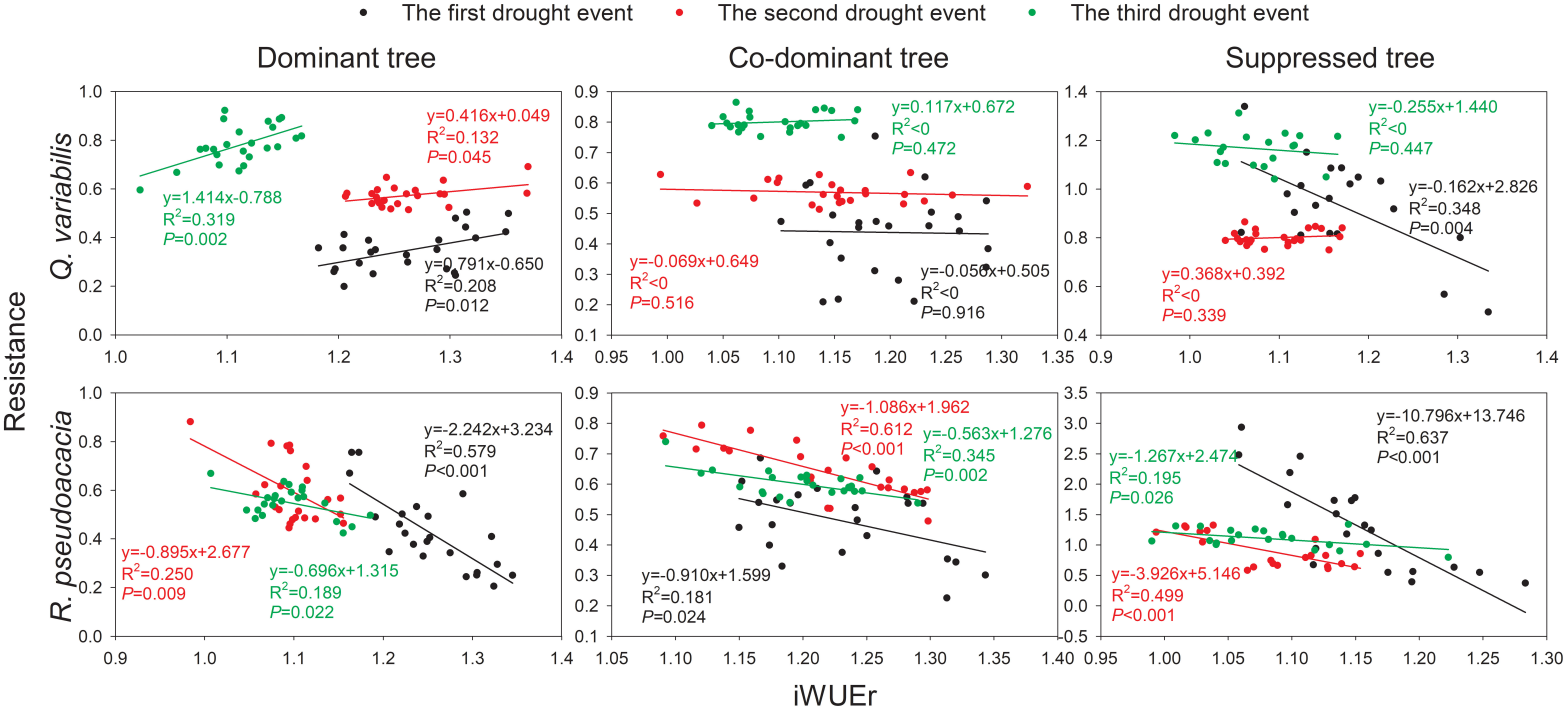
Relationship between Rt and iWUEr of *Q. variabilis* and *R. pseudoacacia* for three canopy classes during drought events.

For *Q. variabilis*, the relationships between Rt and iWUEr of D showed significant positive trends (*p*< 0.05) during the three drought events (**Figure 5**). In contrast, no significant trends of Rt-iWUEr were observed in the CD and S of *Q. variabilis*, except for S in the 2002 drought event (*p* = 0.004). For *R. pseudoacacia*, the Rt-iWUEr of the three canopy classes showed significant negative trends during the three drought events (*p*< 0.05). The negative correlation between Rt and iWUEr eased as the slopes of the regression lines increased with an increase in drought events experienced by trees.

### Growth resilience indices, iWUEr and canopy health

**Figure 6:**
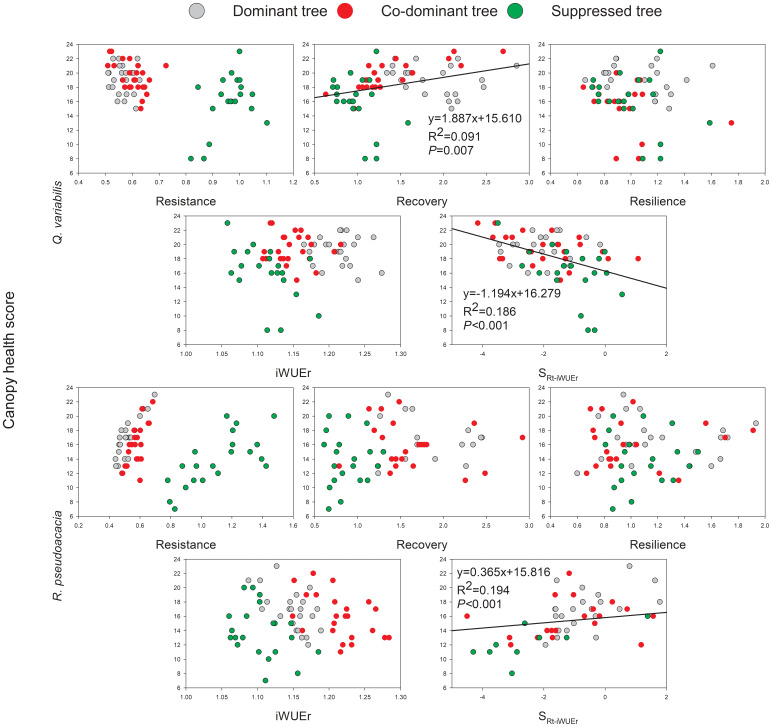
Relationship between canopy health scores and growth resilience indices, iWUEr, and SRt-iWUEr of *Q. variabilis* and *R. pseudoacacia*.

The significant trends were observed between canopy health scores and Rc and S_Rt-iWUEr_ of *Q. variabilis*, on the other hand, health scores increased with S_Rt-iWUEr_ of *R. pseudoacacia* (**Figure 6**), indicating that lower Rc and positive S_Rt-iWUEr_ *Q. variabilis*, negative S_Rt-iWUEr_ *R. pseudoacacia* were the more vulnerable to canopy die-back during drought. The canopy health score as a function of crown class was analyzed using ANOVA, and the results showed that S trees for these two species were significantly more susceptible to canopy die-back than D and CD trees.

## Discussion

Despite receiving far less scholarly attention than natural forests do, plantations play an essential role in rehabilitating endangered ecosystems (Brockerhoff et al., 2008; Rodriguez-Vallejo et al., 2021). By combining resistance indices, iWUE, and canopy health score data, we have provided valuable field data to help predict tree mortality risk in these forests.

### Effects of species and crown classes on BAI and iWUE response to drought

Similar to previous observations, the radial growth of both species was significantly reduced under drought-induced water stress (Martín-Benito et al., 2008; Eilmann and Rigling, 2012; Mazza et al., 2021). A decline in the carbon stock available for functions during drought events is associated with reducing photosynthetic products and changing the method of carbon allocation and utilization (Flexas et al., 2006; Körner, 2015). For example, drought promotes more carbohydrates and starches in tree leaves to be decomposed to regulate osmotic pressure (Chaves et al., 2003)e, and some studies have also shown that under drought, trees may accumulate a larger proportion of non-structural carbohydrates through photosynthesis, which is used to maintain respiration in the dormant season and the growth of earlywood in the following year, which leads to the hysteresis of growth (Lacointe, 2000; Richburg, 2005).

D and CD trees showed higher growth reductions than S trees for both species during drought, whereas the recovery of D and CD was enhanced afterward, consistent with some of the existing studies (Martín-Benito et al., 2008; Merlin et al., 2015; Trouvé et al., 2017). One explanation could be that D and CD trees are more exposed to drought as they intercept more resources in forests dominated by asymmetrical competition (Linares et al., 2010). In addition, despite the general drought conditions, S trees may benefit from better growth conditions (slightly cooler, less radiation, or better ventilation) than D and CD trees because they have less leaf area exposed to intense radiation and high temperatures owing to partial shading (Aussenac, 2000; von Arx et al., 2012). Other features of S trees, including smaller crowns, lower root densities (Liu and Muller, 1993; Augspurger and Bartlett, 2003), and lower carbohydrate reserves (Richardson et al., 2013) may hinder their recovery to pre-drought growth levels (Galiano et al., 2011; Grote et al., 2016).

Accumulated stresses from multiple drought events led to an increase in Rt in *Q. variabilis* and a decrease in Rc in *R. pseudoacacia*. *R. pseudoacacia*, as an early successional species, seems to be maladjusted to frequent drought years typical of low altitudes in North China (Tanaka-Oda et al., 2010). Its poor performance in recovering growth after drought might be due to its ineffective control mechanism in dealing with water loss (Eilmann and Rigling, 2012). We anticipated that given the different levels of drought tolerance among the species, the growth releases would come from the death of the less drought-resistant *R. pseudoacacia*, benefiting *Q. variabilis* radial growth during the upcoming drought event (Rubio-Cuadrado et al., 2018).

Rs measures the capacity of trees to recover their pre-drought growth rate after drought, with Rs< 1 indicating a persistently detrimental effect caused by drought on subsequent growth. With an increase in drought events experienced by trees, the Rs of D showed increasing trends, and the Rs of CD and S showed decreasing trends. This may indicate that the sensitivity of the drought response changed after withstanding numerous drought occurrences. CD and S will be more vulnerable to drought than D, and are more likely to experience carbon famine (Trifil et al., 2017), increasing the likelihood that they will die.

*R. pseudoacacia*, a non-native tree species, had slightly higher iWUE than *Q. variabilis* (a native tree species). Similar results have been reported for other native and nonnative tree species. For example, Song et al. (2019) reported a higher increase in iWUE for non-native trees than for native tree species in forests in Northeast China. When drought occurs, a higher increase in the iWUE for the non-native species compared with the native species seems to be attributable to higher climatic sensitivity or corresponding stronger water stress. In contrast, *R. pseudoacacia* has more conservative water use (Fan, 2010; Nadal-Sala et al., 2017), which partly reflects the differences in xylem structure. *R. pseudoacacia* produces more vessels, but its diameter appears to be smaller than that of oaks (Nola et al., 2020; Özden Keleş and Savaci, 2021). Greater resistance to water transportation and cavitation under drought conditions may be achieved with vessels of smaller diameters (Sperry et al., 2006), resulting in lower hydraulic conductivity and higher iWUE. In addition, under drought conditions, *R. pseudoacacia* closes its stomata at an early stage compared to *Q. variabilis*, resulting in a rapid decline in stomatal conductance and photosynthesis (Fan, 2010; Jin et al., 2011; Colangelo et al., 2018), at the same time, more apparent morphological changes in adaptation to water limitation were observed in *R. pseudoacacia*, such as leaf shedding (Wang et al., 2017), which could partially explain the higher iWUE to drought observed in *R. pseudoacacia*.

S trees were below the main canopy, resulting in less coupling with the atmosphere, which created a higher ratio between boundary layer conductance and canopy stomatal conductance (Wullschleger et al., 2000), as well as assimilated respired CO_2_ (lower δ^13^C) within the canopy, and drought induced stomatal closure. Therefore, reduced mesophyll conductance (g_m_) could increase iWUE. Height alone is supposed to make water transport to the leaves more difficult, resulting in a decrease in g_m_ with height (Cano et al., 2013); the lower the height of S trees compared to D and CD trees, the lower the g_m_. These features may lead to lower iWUE for S trees.

### Drought modifies Rt-iWUEr

In the lithoid hilly areas with shallow soil, the negative trend between Rt and iWUEr could be the result of a more substantial reduction in soil moisture, which causes plants to close their stomata to save water and maintain their leaf water potential within a safe range at the expense of reduced carbon uptake (McDowell, 2011). Unlike *R. pseudoacacia*, *Q. variabilis* showed no significant negative trend in Rt-iWUEr, which agrees with previous results on contrastive research of native and non-native tree species (Song et al., 2019). Non-native tree species may have greater stomatal control than native species, consequently enhancing iWUE to the detriment of growth (Gentilesca et al., 2021). In addition, the absence of Rt-iWUEr might be attributed to variable allocation to other tissues, and remobilization of carbohydrate reserves frequently causes a disconnect between tree growth and carbon assimilation (Urrutia-Jalabert et al., 2015).

### Canopy health

The difference in relationships between canopy health scores, growth resilience indices, and iWUEr of *R. pseudoacacia* and *Q. variabilis* revealed partly different reasons for drought-induced mortality. For *R. pseudoacacia*, SRt-iWUEr was negatively linked to canopy health scores, suggesting that carbon starvation may cause canopy die-back in trees undergoing severe drought (Wang et al., 2017; Colangelo et al., 2018). The lower S_Rt-iWUEr_ indicates that the increased iWUE resulting from multiple drought events prevented carbon uptake by *R. pseudoacacia*. However, reduced carbon uptake has a negative effect on driving phloem transport, maintaining turgor, and refilling embolized xylem during drought, and mortality occurs as soon as one or more of these processes reaches a threshold (McDowell et al., 2011). *R. pseudoacacia* showed a growth reduction in drought-induced mortality at a local scale (Wang et al., 2017). According to their findings, no significant difference was observed in the growth rate between dead and healthy *R. pseudoacacia* before severe drought. After multiple drought events, the growth rate of dying trees fell and remained very low, even resulting in only a row of earlywood conduits without latewood being observed on tree cores during drought events (Wang et al., 2017). Current photosynthetic products are not readily available because of the influence of the main canopy on the photosynthetic capacity of the S trees (Martín-Benito et al., 2008). Structural growth is associated with higher demands for older carbon pools, which are primarily reserves accumulated during favorable growth years. However, replenishing older carbon pools with new sugar inputs may become more challenging after multiple drought events (Richardson et al., 2013). Therefore, for *R. pseudoacacia*, the lower carbon storage and S_Rt-iWUEr_ of S trees compared with those of D and CD trees may explain their higher mortality.

There was a clear link between low Rc capacity and high S_Rt-iWUEr_ to drought in *Q. variabilis* and the increased risk of canopy die-back. Previous studies have revealed a typical pattern for oaks experiencing multiple drought events: oaks usually die a few years after the drought because the time period needed for oak reserves to recover may be too long, resulting in a gradual decline in resilience and increased mortality rates (Galiano et al., 2012; Granda et al., 2013; Granda et al., 2014). For *Q. variabilis*, a higher percentage of consumption of stored nonstructural carbohydrates during drought events resulted in a positive trend in Rt-iWUEr (higher S_Rt-iWUEr_), further limiting the ability of trees to recover following drought occurrences (Galiano et al., 2012; Rosas et al., 2013). No evidence of such depletion of individuals was found in this study, but inconsistent findings were found between net ecosystem productivity based on eddy covariance and *Q. variabilis* biomass growth based on biometric methods in a local mixed deciduous plantation dominated by *Q. variabilis* during drought events, partly because of the consumption of stored non-structural carbohydrates (Jia et al., 2022).

## Conclusion

The effect of drought on the growth and functioning ofin plantations has been anone of the important concerns for researchers and forest managers to proactively address climate-related global challenges. Our study confirms the differing responses of three crown classes, R. pseudoacacia, and Q. variabilis, to several specific drought events.: During the drought, D and CD showed a higher reduction in growth and higher increase amplitude in iWUE than S, did but also recovered faster afterward. A more conservative water-saving strategy was reflected in the negative correlation between Rt and iWUEr inof R. pseudoacacia than in Q. variabilis. Lower Rc, and positive SRt-iWUEr Q. variabilis, and negative SRt-iWUEr R. pseudoacacia were more susceptible to canopy die-back during drought due to stress from multiple drought events. This study provides helpful information on species selection and management measures for plantations in lithoid mountainous areas with increasing drought risk.

## Data availability statement

The original contributions presented in the study are included in the article/**Supplementary Material**. Further inquiries can be directed to the corresponding author.

## Author contributions

HJ and CG contributed to conceptualization, chronology data analysis and wrote the manuscript. CH contributed to stable C isotopes, canopy health score data analysis. JZ, CY, PM contributed to design of the study and funded the study.

## Funding

This work was supported by a National Nonprofit Institute Research Grant of the Chinese Academy of Forestry (CAFYBB2018ZA001) and the National Key Research and Development Project (2020YFA0608101). The China Meteorological Data Service Center provided meteorological data.

## Conflict of interest

The authors declare that the research was conducted in the absence of any commercial or financial relationships that could be construed as a potential conflict of interest.

## Publisher’s note

All claims expressed in this article are solely those of the authors and do not necessarily represent those of their affiliated organizations, or those of the publisher, the editors and the reviewers. Any product that may be evaluated in this article, or claim that may be made by its manufacturer, is not guaranteed or endorsed by the publisher.

## Abbreviations

AC, Autocorrelation; BAI, Basal area increment; CD, Co-dominant; D, Dominant; DBH, Diameter at breast height; EPS, Expressed population signal; iWUE, intrinsic water-use efficiency; MS, Mean sensitivity; S, Suppressed; SPEI, Standardized precipitation evapotranspiration index.
