# Supplementary material for: Drought effects on tree growth, water use efficiency, vulnerability and canopy health of Quercus variabilis-Robinia pseudoacacia mixed plantation
Source: Front Plant Sci. 2022 Oct 12;13:1018405. doi: 10.3389/fpls.2022.1018405 (PMC9597382; doi:10.3389/fpls.2022.1018405)
Supplement: Supplementary file 1 [file DataSheet_1.docx]

**Supplementary material**

**Table S1**. The basic situation of the study plots

| Plot | Latitude | Longitude | Density | Altitude | Slope （°） | Aspect |
| --- | --- | --- | --- | --- | --- | --- |
|  |  |  | （t/ha） | （m） |  |  |
| 1 | N35°02’49.09’’ | E112°28’11.38’’ | 844 | 467 | 3 | S |
| 2 | N35°02’41.44’’ | E112°27’57.98’’ | 911 | 453 | 5 | S |
| 3 | N35°01’52.38’’ | E112°28’18.26’’ | 855 | 432 | 8 | WS |
| 4 | N35°02’52.77’’ | E112°23’17.86’’ | 1422 | 448 | 4 | S |
| 5 | N35°01’52.40’’ | E112°28’18.28’’ | 1300 | 423 | 5 | WS |
| 6 | N35°02’41.86’’ | E112°27’46.60’’ | 1377 | 435 | 7 | N |
| 7 | N35°03’6.41’’ | E112°25’23.30’’ | 633 | 442 | 8 | S |
| 8 | N35°02’56.43’’ | E112°25’38.31’’ | 577 | 421 | 4 | WS |
| 9 | N35°00’55.04’’ | E112°16’51.12’’ | 477 | 413 | 3 | ES |
| 10 | N35°00’51.25’’ | E112°16’41.33’’ | 422 | 415 | 3 | ES |
| 11 | N35°00’52.06’’ | E112°16’47.32’’ | 444 | 413 | 2 | ES |
| 12 | N35°02’1.27’’ | E112°28’37.27’’ | 700 | 421 | 6 | N |
| 13 | N35°05’12.12’’ | E112°27’12.41’’ | 688 | 456 | 6 | S |
| 14 | N35°04’2.09’’ | E112°28’27.19’’ | 744 | 442 | 7 | S |
| 15 | N35°03’2.10’’ | E112°28’27.31’’ | 766 | 436 | 8 | WN |
| 16 | N35°03’16.23’’ | E112°27’27.31’’ | 911 | 441 | 7 | S |
| 17 | N35°04’12.02’’ | E112°27’18.28’’ | 900 | 462 | 9 | S |
| 18 | N35°03’2.33’’ | E112°28’01.33’’ | 1033 | 432 | 6 | WN |
| 19 | N35°05’2.09’’ | E112°27’27.09’’ | 966 | 441 | 9 | ES |
| 20 | N35°01’37.31’’ | E112°28’15.84’’ | 1355 | 415 | 3 | E |
| 21 | N35°01’50.66’’ | E112°27’53.85’’ | 1477 | 423 | 6 | WS |
| 22 | N35°05’50.27’’ | E112°27’23.06’’ | 1244 | 417 | 5 | E |
| 23 | N35°04’23.68’’ | E112°27’33.27’’ | 1322 | 422 | 4 | E |

**Table S2**. Scoring system from Stone et al. (2008) and Nolan et al. (2008)

| Score | Brief description | Expanded description |
| --- | --- | --- |
| Crown size | | |
| 5 | Large, vigorous | Well-balanced, fully-extended crown, shaped by large branches containing a healthy 'hierarchy' of smaller branches supporting foliage |
| 3 | Moderate | Moderately-contracted crown, non-uniform in shape with foliage unevenly distributed. Approximately half of the outer, smaller branches dead or missing |
| 1 | Contracted | Crown contracted, all outer branches dead or missing, foliage on only major branches or stem arising from epicormic growth |
| Crown density | | |
| 5 | Very dense | Very dense leaf clumps with even distribution of clumps over the crown. Very little light penetrating the leaf clumps |
| 4 | Dense | Dense leaf clumps distributed unevenly over the crown |
| 3 | Moderate | Clumps of average density with reasonable distribution or dense clumps very unevenly spread |
| 2 | Sparse | Clumps are sparse and poorly spread |
| 1 | Very sparse | Very few leaves anywhere in crown |
| Dead branches | | |
| 5 | None | No visible dead branches or branchlets/shoots in the crown |
| 4 | Dead terminal shoots | On close inspection some dead terminal branches are evident but not over all the crown |
| 3 | Dead small branches | Some small branches are dead but not over all the crown. These are easily observed but do not give the impression of seriously affecting the crown |
| 2 | Dead main branches | Some large and or small branches dead over part of the crown with the obvious impression of serious branch death |
| 1 | Dead main branches | Large and small branches dead over most of the crown which is obviously dying |
| Crown epicormic growth | | |
| 5 | None | 0–20% of canopy is epicormic in origin |
| 4 | Minor | 20–40% of canopy is epicormic in origin |
| 3 | Moderate | 40–60% of canopy is epicormic in origin |
| 2 | Extensive | 60–80% of canopy is epicormic in origin |
| 1 | Severe | 80–100% of canopy is epicormic in origin |
| Leaf discolouration | | |
| 5 | None | No visible discolouration or browning |
| 4 | Minor | 0–10% of canopy exhibits leaf browning and/or discolouration |
| 3 | Moderate | 10–50% of canopy exhibits leaf browning and/or discolouration |
| 2 | Extensive | 50–100% of canopy exhibits leaf browning and/or discolouration |
| 1 | Severe | All of canopy is brown |
